# Use of a Glycolipid Inhibitor to Ameliorate Renal Cancer in a Mouse Model

**DOI:** 10.1371/journal.pone.0063726

**Published:** 2013-05-09

**Authors:** Subroto Chatterjee, Nezar Alsaeedi, Jennifer Hou, Veera Venkata Ratnam Bandaru, Lan Wu, Marc K. Halushka, Roberto Pili, Georges Ndikuyeze, Norman J. Haughey

**Affiliations:** 1 Department of Pediatrics, Division of Pediatric Cardiology, The Johns Hopkins Medical Institutions, Baltimore, Maryland, United States of America; 2 Department of Neurology, The Johns Hopkins Medical Institutions, Baltimore, Maryland, United States of America; 3 Department of Pathology, The Johns Hopkins Medical Institutions, Baltimore, Maryland, United States of America; 4 Department of Medicine, The Roswell Park Cancer Institute, Buffalo, New York, United States of America; 5 Department of Oncology, The Johns Hopkins Medical Institutions, Baltimore, Maryland, United States of America; University of Alabama at Birmingham, United States of America

## Abstract

In a xenograft model wherein, live renal cancer cells were implanted under the kidney capsule in mice, revealed a 30-fold increase in tumor volume over a period of 26 days and this was accompanied with a 32-fold increase in the level of lactosylceramide (LacCer). Mice fed D- threo-1-phenyl-2-decanoylamino-3-morpholino-1-propanol (D-PDMP), an inhibitor of glucosylceramide synthase and lactosylceramide synthase (LCS: β-1,4-GalT-V), showed marked reduction in tumor volume. This was accompanied by a decrease in the mass of lactosylceramide and an increase in glucosylceramide (GlcCer) level. Mechanistic studies revealed that D-PDMP inhibited cell proliferation and angiogenesis by inhibiting p44MAPK, p-AKT-1 pathway and mammalian target for rapamycin (mTOR). By linking glycosphingolipid synthesis with tumor growth, renal cancer progression and regression can be evaluated. Thus inhibiting glycosphingolipid synthesis can be a bonafide target to prevent the progression of other types of cancer.

## Introduction

Recent studies suggest that sphingolipids can induce phenotypes such as proliferation, adhesion and angiogenesis-the hallmarks in tumor growth and metastasis [1]–[6]. Application of drugs which inhibit glycosphingolipid synthesis provide an opportunity to examine the role of these compounds in animal models of human disease. Here we demonstrate that by linking glycosphingolipid synthesis and its inhibition in a mouse model of renal cancer, it is possible to observe the footprint of interactions between drug and glycosphingolipid metabolizing enzymes and to predict the onset of disease/tumor progression and tumor regression. Blocking the glycosylation of ceramide to treat cancer has been documented in cell and in animal models [2], [3].

Tumors require new blood vessel formation from pre-existing ones (angiogenesis) and vascular endothelial growth factor (VEGF) plays a critical role in inducing angiogenesis in a variety of tumors [4], [5]. Therefore, we rationalized that targeting endothelial cells that line the tumor blood vessels, which are enriched with one isoform of LCS can have several theoretical advantages such as targeting drug delivery in several types of cancer. Our rationale stems from our previous findings wherein VEGF was shown to stimulate LCS: β-1,4-GalT-V activity in human arterial endothelial cells to produce LacCer, and that resulted in the activation of the “oxygen sensitive” signaling pathway leading to angiogenesis [4]. Furthermore, the use of small interfering RNA (siRNA) to cause LCS: β-1,4-GalT-V gene ablation or pharmacological manipulation by the use of D-PDMP, an inhibitor of uridine diphosphate glucose ceramide glucosyltransferase (UGCG), and LCS [7], markedly mitigated VEGF-induced angiogenesis [5]. Also, we observed that D-PDMP can significantly (*P*<0.0005) mitigate VEGF/β-FGF induced angiogenesis *in vivo* in nude mice [4] and inhibit experimental metastases of murine Lewis lung carcinoma [8].

The aim of this study was to determine whether inhibiting glycosphingolipid synthesis would also inhibit cell proliferation/reduce tumor volume *in vitro* and *in vivo*. This study achieved the aim that inhibiting glycosphingolipid synthesis would also inhibit cell proliferation/reduce tumor volume *in vitro* and *in vivo*.

## Materials and Methods

### Ethics Statement

This study was approved by The Johns Hopkins Medicine Institutional Animal Care and Use Committee, permit #MO03M615. The relevant efforts taken to ameliorate animal suffering including anesthesia (Ketamine and Xylazine) and post-surgery follow-up (periodical check on animals for up to 8 hrs post-surgery). Antibiotic ointment was administered at site of incision and an optical ointment was applied to prevent their eyes from drying out during surgery.

### Experiments with mice

The mouse renal cancer (RENCA) cell suspension was loaded into a 1 ml syringe. Recipient mice (BALB/c mice) were pre-anesthetized with i.p. injection (100 uL) of a stock solution of ketamine/xylazine (8 mg/kg), secured on a grounded plate with the back skin shaved and prepared in a sterile fashion. An incision was made in the left flank and the left kidney and exteriorized. 100 uL of tumor cell suspension (1×10^6^ cells/inoculum) was injected in the subcapsular space of the left kidney. The kidney was then placed in the original position and the incision was closed with sterile metal secure clips. Experimental treatment began 2 days after subcapsular tumor implantation. Mice were fed by oral gavage D-PDMP (3 and 10 mg/kg body weight) daily. D-PDMP was solubilized in 5% Tween-80 in saline. Ten mice (N = 10) were used in each group.

The placebo group of mice having the tumor implant received daily, an equal volume of 100 uL of vehicle. After this procedure, animals were monitored daily. End point of this set of experiments was tumor growth assessment in the kidney. Tumor growth monitoring in animals implanted orthotopically in the kidney was performed by manual palpation twice a week. After 4 weeks, (day 26–28 post- tumor implantation), animals were euthanized with CO_2_ and autopsied. Tumor growth measurement was performed by direct tumor weight assessment at the end of the experiment.

### Histological studies

Primary tumors were sent for histology (N = 4). The tissue sections were stained with hematoxylin –eosin and with antibodies against CD31 (an important marker for angiogenesis) and caspase-3 (an important marker for apoptosis). Lactosylceramide antibody (CD17) was purchased from Ancell Laboratories and was used to identify the localization of LacCer in the kidney tissue. The tissues were harvested from the other 4 mice and used in Biochemical/molecular assays described in the text.

### Extraction of D-PDMP and sphingolipids

Sample extraction was conducted using a modified Bligh and Dyer procedure as previously described [9]. Each sample was homogenized at room temperature in 10 volumes of deionized water. Three volumes of 100% CH_3_OH containing 53 mM HCOONH_4_, 17 ng/ml C_12∶0_ ceramide, and 17 ng/ml C_12∶0_ sphingomyelin (internal standards, IS) was added, and the mixture was vortexed. Four volumes of CHCl_3_ were added, vortexed, and then centrifuged at 1000×*g* for 10 minutes. 100 µl of the CHCl_3_ layer was separated, dried under a stream of nitrogen, and the residue was dissolved in 100% methanol containing 10 ng/ml C_2∶0_ ceramide as an internal standard for quantification of D-PDMP. Then the samples were transferred into glass vial inserts for LC/MS/MS analysis; 10 µl was injected into the LC/MS/MS. All solvents and chemicals were HPLC grade. Lipid extractions were performed using borosilicate-coated glass tubes and pipettes, to reduce adhesion of lipids to surfaces.

### LC/MS/MS analysis for sphingolipids and D-PDMP

Identification of sphingolipid species and D-PDMP was performed using a 3000 PE Sciex liquid chromatography electrospray ionization tandem mass spectrometer, operated in positive mode (LC-ESI/MS/MS; Applied Biosystems, Thornhill, Ontario, Canada), and according to methods reported in previous studies [10]. The system consists of two Perkin Elmer LC pumps connected to a HTC PAL auto-injector (CTC Analysis, Zwingen, Switzerland) fitted with a 50 µl sample loop. Flow rate was 400 µl/min for ceramides and 1 ml/min for sphingomyelins. Flow from the sample injector led to a 2.6 µm C column for ceramides and D-PDMP, and a 5 µm C_18_ column for sphingomyelins (Phenomenex, Torrance, CA). The column was pre-equilibrated for 0.1 min with solvent A that consisted of CH_3_OH:dH_2_O (85∶15, v/v) with 5 mM HCO_2_NH_4_, and the sample was eluted with solvent B that consisted of CH_3_OH:HCOOH (99∶1, v/v) with 5 mM HCO_2_NH_4_. The eluted sample was automatically introduced into the ion source. Instrument parameters for LC-ESI/MS/MS were optimized individually for each species of ceramide, ceramide-1-phosphate, sphingosine, sphinganine, sphigosine-1-phosphate, sphingomyelin, and D-PDMP by multiple reaction monitoring. Ceramides and sphingomyelins were quantified by area under the curve (AUC) of counts per second (CPS) normalized to internal standards (ceramide C_12∶0_ and sphingomyelin C_12∶0_). D-PDMP concentrations were determined by creating a standard curve that was constructed using the ratios of reference standards (D-PDMP; 0.1–100 µg/ml) to IS (ceramide C_2∶0_; 10 ng/ml) plotted against the ratios of reference and IS from experimental samples. All stock solutions were stored at −20°C and stable over 6 months.

### Glycosyltransferase and glycosylhydrolase activity assays

LCS activity in kidney preparations was measured using a protocol detailed previously [11] with the following modifications. Briefly, flash frozen kidney tissue were homogenized in Tris-HCl buffer (pH 7.4) containing Triton-X-100 and centrifuged at 10,000 rpm for 15 min. The protein mass in the supernatant was measured and 100 µg of sample protein was used in enzymatic assays, in triplicate. 14C-UDP-galactose (American Radiolabel Company, St. Louis, MO) served as the donor of galactose. And glucosylceramide (Matreya Chemicals, PA) served as the acceptor in this assay. 14C-LacCer generated from this assay was quantified by scintillation spectrophotometry. Glucosylceramide synthase activity was measured using ceramide as the acceptor and [3H] UDP- glucose as the glucose donor. Glucosylceramide hydrolase activity was measured using [3H] glucosylceramide (American Radiolabel Company), as the substrate according to previously published protocols.

### Western immunoblot studies

Kidneys were harvested from control, placebo, 3 MPK and 10 MPK of D-PDMP fed orally and daily to mice. The kidney tissues were homogenized in RIPA buffer (Sigma Aldrich), placed on ice for 15 minutes, centrifuged at 13,000 RPM, and the supernatants were collected. To determine the protein concentration, Bradford Assay was used. To a 4–15% gel (Bio-Rad Ready Gel), 45–90 µg of protein samples and 10 µL of low molecular weight protein standard (Bio-Rad Dual Color Precision Plus) were loaded. After gel electrophoresis for 1.5 hrs, Immuno-Blot PVDF membranes (Bio-Rad) were soaked in cold methanol, dH_2_O, and transfer buffer (Bio-Rad). Next, the gel was transferred to a PVDF membrane (Bio-Rad), at 30 V overnight. Membranes were blocked in 3% milk, 1×TBST (0.05% Tween-20), incubated in primary antibodies against: β-1,4-GalT-V, Beta actin, UGCG (Santa Cruz Biotechnology), GAPDH (glyceraldehyde 3-phosphate dehydrogenase, US Biological), p44MAPK (Thr202 and Tyr204), p-AKT-1 (Thr308) (Cell Signaling Technologies), and mTOR (Sigma-Aldrich) in 1×TBS with 1∶200 dilution and 0.5% BSA, and incubated in secondary antibody, goat anti-rabbit IgG HRP conjugate (Sigma Aldrich) in 5% milk, 1×TBST with 1∶1000 dilution. All primary antibodies required overnight incubation at 4°C while secondary antibodies required 1 hr incubation at room temperature. Next, membranes were washed in 1×TBST for 5–10 minutes and repeated 2 times and developed using the ECL kit (Amersham ECL Plus™ Western Blotting Detection Reagents).

### Computer assisted quantitation of caspase-3 and CD31 positive cells

All immunohistochemically stained slides were digitally scanned using an Aperio CS ScanScope (Vista, CA). For Caspase-3 analysis, the rare event and nuclear algorithms of the Aperio Tool Box Kit were utilized. The rare event detector identified caspase positive cells which were confirmed by hand-curation. This value was divided by the total cell count of the area determined by the nuclear algorithm. CD31 area was identified by dividing a CD31 positive mask area by the viable tumor mask area for each slide, as similarly described [12].

### Statistical studies

The data are expressed as a mean ± standard deviation and as a mean ± standard error measurement. The significant difference between the control, placebo, and experimental group was evaluated using the student's *t* test and one way ANOVA. Pair wise comparison was made to determine the significance between different treatments. Differences were considered significant at *P*<0.05.

## Results

We observed that D-PDMP but not L-PDMP inhibited the growth of RENCA cells (See Figure S1). Therefore, we hypothesized that inhibiting LCS activity and reducing LacCer load may well reduce renal cancer in mice. In this study, we used D-PDMP since it is non-toxic and is taken up by the kidney, brain and liver when given orally to mice [13]. Also, it is rapidly cleared by the cytochrome P450 system as its t_1/2_ is ∼50 minutes [13]. Although D-PDMP was synthesized to mitigate Gaucher's disease by inhibiting UGCG activity, we showed that it also inhibited LCS activity-LacCer production and thus angiogenesis *in vitro* and *in vivo*, in nude mice [5]. Herein, we demonstrate that inhibiting LCS activity and LacCer production specifically are central to signaling events such as angiogenesis and proliferation that lead to a marked reduction in tumor volume in a mouse model of renal cancer.

**Figure 1:**
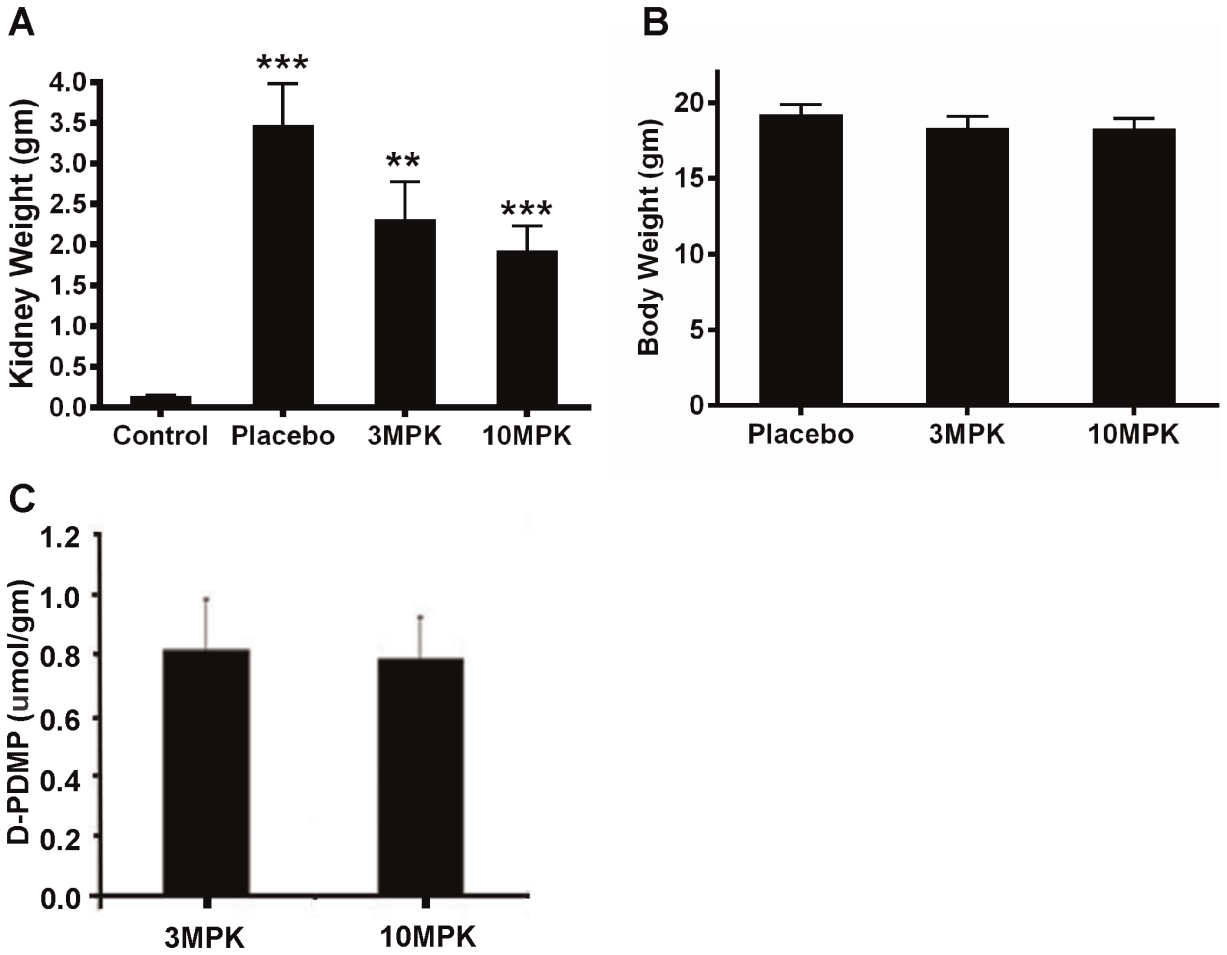
D-PDMP reduced tumor volume in mice. Mice (BALB/C) were pre-anesthetized and RENCA tumor cell suspension (10^6^ cells) was injected in the subcapsular space of the left kidney. Experimental treatment began two days after subcapsular tumor implantation. Mice fed by oral gavage D-PDMP (3 and 10 mg/kg body weight, MPK) daily. D-PDMP was solubilized in 5% Tween-80 in saline. Placebo mice received the vehicle (5% Tween-80 in saline 100 µl only). After 26 days, the mice were sacrificed. The right kidney served as control, and the left kidney served as placebo or drug treated with 3 and 10 MPK of D-PDMP. **A**: A marked increase in mouse kidney tumor volume is sharply decreased by feeding D-PDMP. **B**. D-PDMP (3 and 10 MPK) did not reduce total body weight in mice. **C**: The level of D-PDMP in kidney of mice treated with 3 and 10 MPK of D-PDMP were similar.

**Figure 2:**
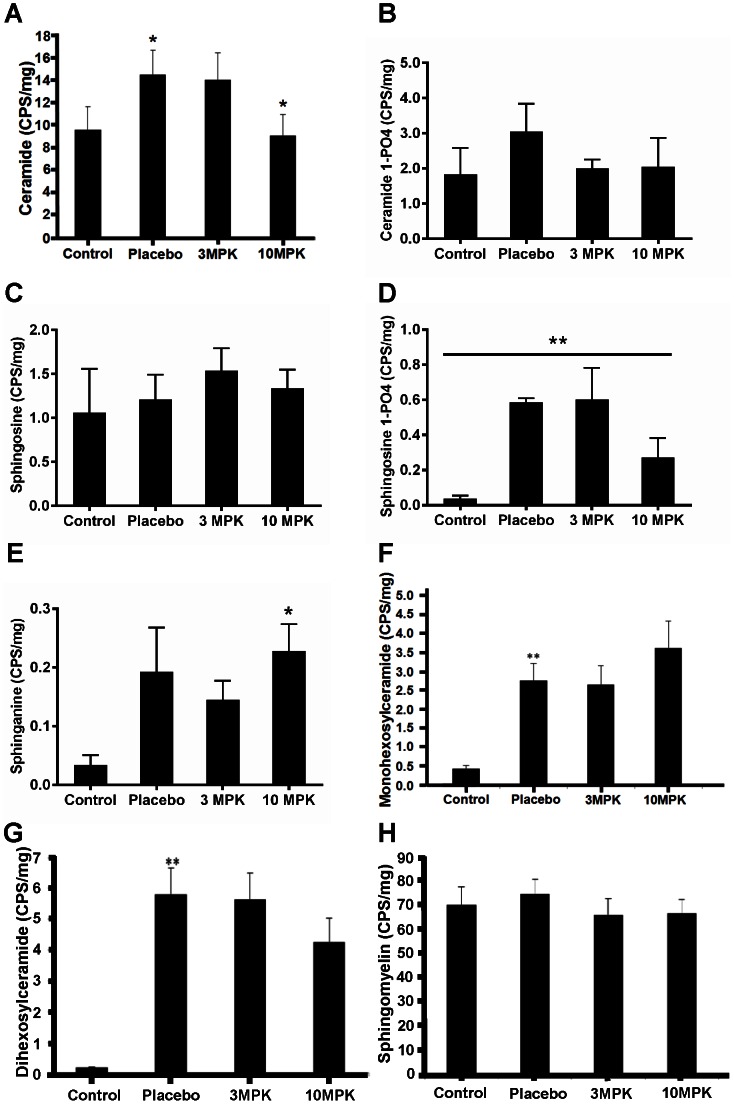
D-PDMP treatment altered the levels of several sphingolipids but not sphingomyelin in mice renal cancer. The level of various sphingolipids were measured by tandem LC-MS/MS. **A**: D-PDMP decreased the level of ceramide, **B**: ceramide-1-phosphate, and **D**: sphingosine-1-phosphate. D-PDMP modestly increased the level of sphingosine; **C**, **E**: sphinganine and **F**: monohexosylceramide. **G**: D-PDMP dose-dependently decreased the level of dihexosylceramide in mice renal cancer. **H**: D-PDMP did not change the level of sphingomyelin in mouse renal cancer. (* p<.05, **p<.01 N = 4).

**Figure 3:**
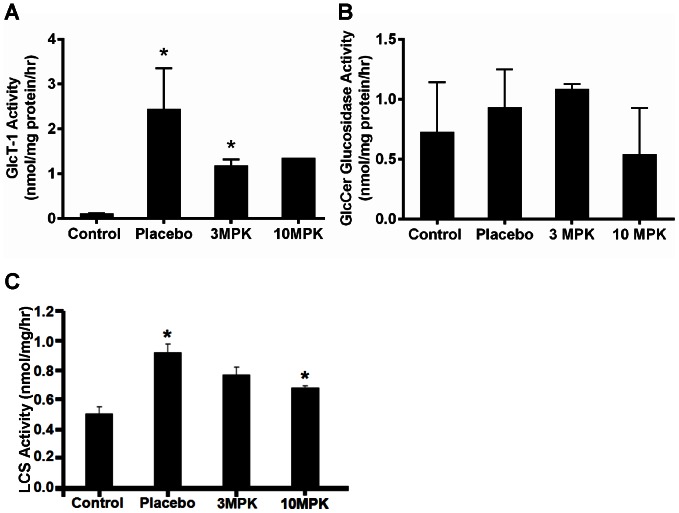
D-PDMP decreased the activity of glycosyltransferases and glycohydrolase in mouse renal cancer. Measurement of lactosylceramide synthase activity in mouse kidney was conducted using UDP[14C] galactose as galactose donor and GlcCer as the acceptor, as detailed [11]. We observed that feeding D-PDMP dose-dependently decreased the activity of this enzyme **A**: glucosylceramide synthase activity was decreased to the same extent irrespective of whether 3 MPK or 10 MPK of D-PDMP was fed to mice with renal cancer, **B**: glucosylceramidehydrolase activity was decreased by D-PDMP treatment and **C**: lactosylceramide synthase activity was dose-dependently decreased in D-PDMP fed mice with renal cancer (N = 4; *p<.05).

One week after application, the primary tumor was macroscopically visible; after 10 days spontaneous metastasis developed in the regional lymph nodes, lung, peritoneum, and liver, allowing the RENCA model to be staged similarly to human renal cell carcinoma. The mean survival time of RENCA-bearing mice is 32 days when 10^6^ RENCA cells are injected. To determine whether alteration in glycosphingolipid profile is an important feature in renal cancer, we compared the level of these lipids in control kidney (not bearing RENCA) versus placebo mice kidney (bearing RENCA). Simultaneously, we examined the effects of feeding D-PDMP on tumor volume and lipid levels in kidney tissue. We observed that implantation of RENCA cells in the subcapsular space in the left kidney (placebo mice, and no treatment) contributed to ∼30-fold increase in tumor volume, over a period of ∼26 days (Figure 1A). The body weight did not change (Figure 1B) and 20% of mice in the placebo group died. Oral feeding of 3 MPK D-PDMP and 10 MPK D-PDMP for the experimental period reduced the tumor volume to ∼50% that of placebo mouse kidney tumors. This D-PDMP dose-independent decrease in tumor volume could, in part, be explained by our observation that the level of D-PDMP (measured using LC-MS/MS) in the kidney tissue was similar (Figure 1C) as it may have been rapidly excreted. Detailed tandem LC-MS/MS revealed that C24, C16 and C22 were the major fatty acid molecular species in ceramide, mono and di hexosylceramide and sphingomyelin in control, placebo, and D-PDMP-fed mice kidney tissue. And C20, C18 and C26 were the minor fatty acid containing sphingolipids (Figure S2). The total ceramide level increased ∼2.5–fold in placebo mouse kidney vs. control. Interestingly, feeding 3 MPK D-PDMP did not change the level of ceramide in kidney compared to placebo (Figure 2A). However, in 10 MPK D-PDMP fed mice, the level of ceramide decreased to control level. Thus, although D-PDMP is claimed to be a specific inhibitor of UGCG and thus contributing to an increase in ceramide levels, in this study it did not raise the tissue levels of ceramide in mouse kidney. And this observation is in agreement with a previous report that showed that D-PDMP (100 MPK) decreased kidney ceramide level∼9% 5 hrs later [13]. Injecting 40 MPK D-PDMP twice a day for 4 days also resulted in a 23% decrease in ceramide in kidney [13]. In another study using the iminosugar N-(5-adamantinane-1′-yl-methoxy)pentyl-1-deoxynoijirimycin(AMP-DNM), an inhibitor of UGCG also did not raise ceramide levels in liver and maintained the plasma level of ceramide to basal level in diet-induced hyperlipidemia in apoE−/− mice [14]. The reasons for a D-PDMP mediated decrease in ceramide level in mouse kidney is not clear from this study. Since ceramide is a direct precursor of sphingomyelin synthesis, we anticipated a higher level of sphingomyelin in D-PDMP treated mice. However, the level of sphingomyelin in our study and in a previous study [13] did not change significantly (Figure 2H). This could be because while a higher pool of ceramide could induce an increase in sphingomyelin synthesis; however, since D-PDMP also increases the activity of sphingomyelinase, it may help maintain sphingomyelin homeostasis [13]. Ceramide could also be hydrolyzed to generate sphingosine and its derivatives (Figure 2C–E). For example, our additional studies reveal that the level of ceramide-1-phosphate (Figure 2B), sphingosine (Figure 2C), sphingosine-1-phosphate (Figure 2D) and sphinganine (Figure 2E) varied in placebo mouse kidney compared to control and upon treatment with D-PDMP. In particular, 20-fold increase in the level of sphingosine-1-phosphate in placebo mouse versus control was noted. Treatment with 3 MPK D-PDMP did not decrease the level of sphingosine-1-phosphate compared to placebo mouse kidney. However, upon feeding 10 MPK D-PDMP, we observed a 50% decrease in the level of this lipid. The level of monohexosylceramides was elevated 5-fold in placebo mouse as compared to that of control mouse kidney but did not change upon feeding 3 MPK D-PDMP (Figure 2F). Surprisingly, the level of this lipid increased somewhat upon feeding 10 MPK of D-PDMP. This observation could be interpreted to indicate a compensatory decrease in the level of LacCer in mice treated with 10 MPK of D-PDMP which decreased the activity of LacCer synthase. Thus, an unexpected result in this study is that long–term (26days) feeding of D-PDMP did not decrease the level of monoglycosylceramides in mouse kidney even though D-PDMP has been previously shown to serve as an inhibitor of glucosylceramide synthesis. The largest increase in the level of dihexosylceramide, ∼32-fold, occurred in placebo kidney as compared to control. However, the level of lactosylceramide decreased dose-dependently in mice fed 3 MPK and 10 MPK of D-PDMP (Figure 2G). This was accompanied by a D-PDMP dose-dependent decrease in the activity of LacCer synthase (Figure 3C) but not GlcCer synthase (Figure 3A). In contrast, we observed that the activity of glucosylceramide hydrolase decreased, with an increase in the dose of D-PDMP from 3 MPK to 10 MPK (Figure 3B). Reduced activity of glucosylceramide glucosidase in mice treated with 10 MPK D-PDMP (Figure 3B) might have contributed to an increased level of GlcCer (Figure 2F). Interestingly, the level of sphingomyelin did not change in control, placebo, and D-PDMP-fed mouse kidney (Figure 2H). In sum, the increase in kidney tumor volume in RENCA bearing mice and its decrease upon treatment with D-PDMP correlated best with the levels of LacCer mass, compared to any other sphingolipid investigated in this study.

Next, using a commercially available monoclonal antibody against LacCer, we determined whether LacCer was a major lipid accumulating in renal cancer. Our immunohistochemical studies revealed the accumulation of large quantities of lactosylceramide within cytoplasmic vesicles (white arrows) exclusively in cancer cells (compare Figure S3A; DAPI stain vs. Figure S3B; CD17 antibody stained cells). Previously, we have shown that in human tumor kidney proximal tubular cells, the activity of LCS is increased, and this is accompanied with an increase in the level of LacCer as compared to normal human kidney [15]. Increased level of LacCer has also been reported in human renal cancer [16]. These findings suggest that in the mouse model of renal cancer, the increase in lactosylceramide mass is best correlated with the increase in the tumor volume and progression of disease. Thus, targeting glycolipid synthesis, in particular LCS and LacCer may be a bonafide therapeutic approach to mitigate renal cancer.

**Figure 4:**
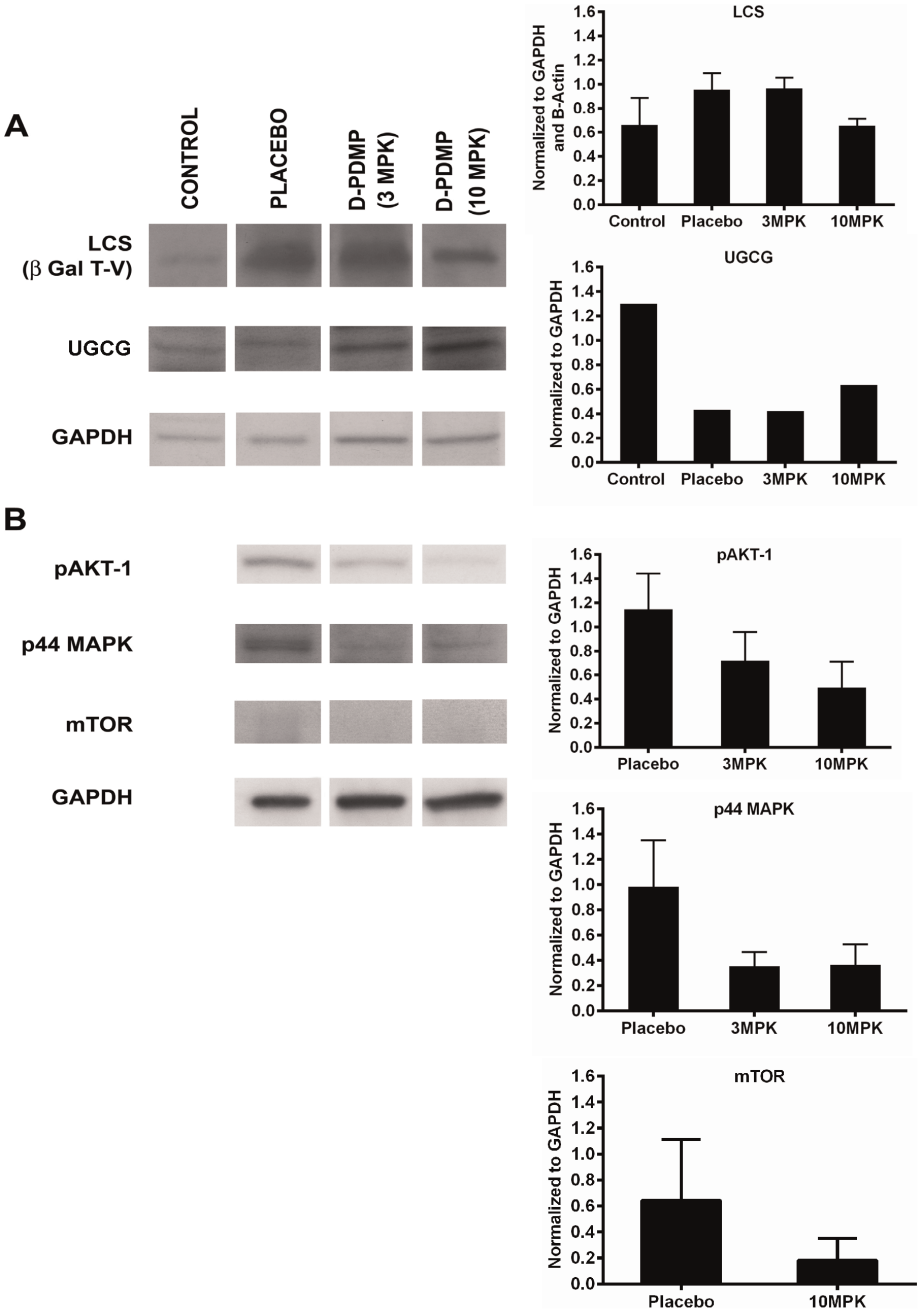
D-PDMP alters the expression of various glycosyltransferases and components of signaling pathways leading to cell proliferation and angiogenesis in a mouse model of renal cancer. Representative immunoblots of tissue extracts from BALB/c mice fed vehicle (placebo plus RENCA cells/tumor) and 3 and 10 MPK of D-PDMP for 26 days and control kidney and corresponding densitometric scans are shown. A: D-PDMP dose dependently decreased the protein expression of LacCer synthase (β-1,4-GalT-V) (N = 3–6), in mouse renal cancer but somewhat increased the protein expression of UGCG (N = 2) in mouse renal cancer. B: Western immunoblots of markers for cell proliferation (p44MAPK, N = 3), angiogenesis (pAKT-1, N = 3; mTOR, N = 3 for placebo and 10 MPK) and house keeping protein GAPDH. D-PDMP treatment reduced the markers for cell proliferation and angiogenesis. Data were assessed by one-way ANOVA.

To understand the molecular pathways contributing to a reduction in tumor volume due to D-PDMP feeding in mice, we conducted western immunoblot assays of several biomarkers, shown by others and us [5], [17], [18], [19] to contribute to cell proliferation, angiogenesis, and apoptosis. Our mechanistic studies revealed that D-PDMP affected a marked reduction in tumor volume by decreasing the expression of various signaling molecules implicated in the pathways contributing to cell proliferation and angiogenesis. For example, there was a marked increase in the mass of LCS in placebo mouse kidney compared to that of control and this observation may explain a marked increase in LacCer level in placebo kidney vs control. D-PDMP dose-dependently decreased the mass of LCS (Figure 4A). In contrast, the level of UGCG was increased in D-PDMP fed mice (Figure 4A). Biomarkers for cell proliferation and angiogenesis e.g. p44MAPK and p-AKT-1 were all decreased in kidney of mice fed D-PDMP compared to that of placebo kidney (Figure 4B). These observations suggest that D-PDMP affects cell proliferation and angiogenesis by inhibiting the activity of LCS and LacCer production. In parallel *in vitro* studies, we have observed that D-PDMP but not L-PDMP dose-dependently decreased the proliferation of RENCA cells, possibly due to the arrest of cells in the G2–M phase of the cell cycle, upon treatment with D-PDMP [20] (Figure S1).

Histological evaluation of the kidneys revealed extensive growth of aggressive RENCA, with marked necrosis. Necrosis, as a percent of tumor volume was quantified using digital analysis tools [21] and found to be 38.8%, 28.4% and 33.2% respectively for placebo, 10 MPK, and 25 MPK treated mice (Figure S4A–H). We have previously shown that D-PDMP can effectively inhibit VEGF–induced angiogenesis *in vitro* in human umbilical vein endothelial cells and human arterial endothelial cells [4], [5]. Also in a previous study, the VEGF+β−FGF induced angiogenesis was mitigated by D-PDMP in mice [4]. This was measured by a marked decrease in the expression of CD31 and angiogenesis. To determine whether a decrease in kidney tumor volume was also due to a decrease in the supply of blood by way of decreased angiogenesis, we performed immunostaining for CD31 (Figure S4I, J). There was a marked decrease in CD31 positive vascular area in viable areas of the tumor (0.3% vs. 0.05% for placebo vs. 25 MPK treated mice) as determined using digital analysis tools [21]. Previously, mTOR has been established as a marker of angiogenesis and has been a validated target to mitigate several types of cancer. We observed that D-PDMP markedly decreased mTOR protein expression (Figure 4B), suggesting that by inhibiting angiogenesis, D-PDMP deprived the tumor of blood supply and thus contributed to a reduction in tumor volume. An unexpected result was that D-PDMP significantly decreased the number of apoptotic cells measured by caspase-3 immunostaining. The percent of caspase positive cells were 0.09%, 0.04%, and 0.03% for placebo, 10 MPK, and 25 MPK treated mice respectively, as determined by digital analysis (Figure S4K–N) [21]. Thus our *in vivo* studies suggest that D-PDMP does not reduce tumor volume by inducing apoptosis in mice kidney.

## Discussion

The following observations may be drawn from our present study implicating the role of glycosphingolipids in renal tumor biology. First, there is a strong and statistically significant correlation between an increase in mouse renal tumor volume and a parallel increase in the mass of LacCer. Second, inhibition of glycosphingolipid glycosyltransferase activity, and particularly the decrease in the activity and mass of LacCer synthase was correlated with a decrease in tumor volume. Third, although D-PDMP is known to be an inhibitor of UGCG, it did not raise the kidney levels of ceramide. Since ceramide is implicated in apoptosis, our studies suggest that D-PDMP does not reduce tumor volume by inducing apoptosis via the ceramide pathway in mice kidney. Rather, D-PDMP inhibited a signaling pathway-induced by LacCer thus contributing to an inhibition of cell proliferation and tumor angiogenesis. Collectively, these studies suggest that the inhibition of glycolipid glycosyltransferase can inhibit proliferation/angiogenesis in tissues via mechanisms independent of apoptosis.

In the present study, a ∼30-fold increase in tumor volume in placebo mouse kidney (compared to that of control) was paralleled by an equal fold increase in LacCer mass. Feeding D-PDMP markedly reduced tumor volume by way of decreasing the enzymatic activity of LCS, LCS mass, and consequently LacCer mass, and the angiogenic proteins such as p-AKT-1 and mTOR. In our previous studies, we observed that the use of siRNA for LCS *in vitro in human endothelial cells* [5] and *in vivo in mouse glioblastoma* [22] and the use of D-PDMP in this study can reduce tumor volume by mitigating angiogenesis. Thus, targeting glycolipid synthesis in general and LacCer synthase in particular [5], [22] is a novel approach to mitigate renal cancer in mice. We have also previously reported that L-PDMP, which activates LacCer synthase in endothelial cells can also induce angiogenensis in a dose-dependent manner [7] and also RENCA cell proliferation in the present study (see Appendix). Thus activation/inactivation of LacCer synthase by agonists/antagonists may well regulate angiogenesis *in vitro* and *in vivo*.

Our studies and those of others [13] have shown that D-PDMP is non-toxic when given at doses ten times that of the concentration used in the present study. The body weight in this study did not differ in placebo vs. D-PDMP–treated mice. The tumor weight decreased approximately 50% in 3 MPK and 10 MPK fed mice compared to placebo. However, when mice were fed higher amounts of D-PDMP; 25 and 50 MPK, it did not further reduce tumor volume (data not shown). In a previous study, it was shown that the t_1/2_ of D-PDMP in mice blood is ∼50 min [13]. Consequently, it is feasible that beyond a threshold of 10 MPK, most of this compound is rapidly removed by excretion and therefore further reduction in tumor volume was not observed. Previously, D-PDMP has been used extensively to examine the role of glycosphingolipid and related glycosytransferases in arterial smooth muscle cell proliferation [1], [23], [24], wound healing [1], [23], [24], osteoclastogenesis [25], polycystic kidney disease [26], [27], elasticity [28], respiratory diseases [29], [30], glioblastoma research [22], [31], [32] cholesterol efflux [33], inflammation *in vitro* [17] and *in vivo* [34], shear stress [35], and A beta secretion in neuroblasotma cells [36].

Although D-PDMP is known to inhibit the activity of UGCG, raise ceramide levels and induce cell death by apoptosis-we could not reproduce these observations *in vivo* in mice kidney. In agreement with a previous study [13] we also observed that the level of ceramide in kidney in D-PDMP –treated mice was lower. Likewise, an iminosugar (AMP-DNM), another inhibitor of UGCG also did not raise the level of ceramide in a transgenic mouse model of hyperlipidemia [14]. Moreover, in a recent study, the use of another glucosylceramide synthase inhibitor, Genz-122346, in a mouse model of polycystic kidney disease revealed that this compound also inhibits proliferation but does not inhibit apoptosis involving ceramide [27]. This could be due to further catabolism of ceramide as the activity of several hydrolases including ceramide deacylase [37] maybe higher upon treatment with D-PDMP. Also ceramide may be converted to other sphingolipids (see above). These observations attest to the multiple fates of ceramide and multiple pools of ceramide in kidney tissue. Indeed, we observed that the activity of GlcCer glucosidase was increased in D-PDMP–treated mice compared to placebo. This may have contributed to an increase in the level of GlcCer in mice fed D-PDMP (10 MPK).

We have previously shown that in cultured human arterial endothelial cells, D-PDMP can inhibit VEGF-induced angiogenesis and this was bypassed by LacCer but not S-1-P. Such observations suggest that LacCer mediated and VEGF-induced angiogenesis is independent of S-1-P-induced angiogenesis [4], [38]. Moreover, use of 1-phenyl-2-palmitoylamino-3-morpholino-1-propanol (PPMP); a relatively more specific inhibitor of GlcCer synthase compared to D-PDMP to mitigate VEGF induced angiogenesis was bypassed by feeding LacCer in human endothelial cells [5]. In fact VEGF-induced angiogenensis in these cells were mitigated ∼1.5 fold better by the use of D-PDMP compared to PPMP. Finally, LCS gene ablation by the use of siRNA mitigated VEGF induced angiogenesis in these cells [5]. In the present study, we document that D-PDMP may well inhibit angiogenesis by way of mitigating the expression of p-AKT-1 and mTOR expression in mice kidney. Collectively, our observations imply that the target of VEGF action is LCS leading to angiogenesis. And the inhibition of LacCer levels due to a decrease in LCS activity and LCS mass upon feeding D-PDMP contributes to the inhibition of angiogenesis and decreased renal tumor volume. In sum, these studies suggest that D-PDMP may be well suited to effectively and safely mitigate tumor growth and also neo-intimal proliferation following balloon angioplasty in rabbits [24] and eventually in man. And this is substantiated from the works conducted in other laboratories wherein D-PDMP was shown to target LCS to mitigate various phenotypes *in vitro* and *in vivo* [25], [28], [34], [39]. Clearly, D-PDMP is not a specific inhibitor of UGCG. Never the less, it is commercially available and its kinetics and bioavailability are known. It is not toxic and is well tolerated by experimental animals. It has been used widely and has increased our knowledge of the inter relationship between glycosphingolipid metabolism and various phenotypes *in vitro* and *in vivo* [22], [24]–[36]. On the other hand, the rapid turnover of D-PDMP requires that some derivative of this compound and/or an alternative approach of its delivery may be relatively more efficacious in mitigating tumor growth and angiogenesis.

## Supporting Information

Figure S1 **Effect of D-PDMP and L-PDMP dose on cell proliferation in mouse renal cancer cells (RENCA).** Cells (×10^3^) were seeded in 96 well sterile trays. When the cells reached ∼80% confluence, they were switched to 2% serum containing medium with various concentrations of D-PDMP and L-PDMP (3H) Thymidine (5 uci/ml). Following incubation for 24 hrs, medium was removed. Cells were washed with PBS (5×) and the incorporation of (3H) Thymidine into DNA was measured as described (N = 12, *p<.05) [12].
(TIFF)

Figure S2 **Fatty acid molecular species involved in lactosylceramide calculations.** Species used for LC/MS calculation of lactosylceramide were 18∶1/16∶0, 18∶1/18∶0, 18∶1/20∶0, 18∶1/22∶0, 18∶1/24∶0, 18∶1/26∶0. Same species were used for ceramide and glucosylceramide. 18∶1/24∶0 was the most elevated species for lactosylceramide calculation. Drug treatments (3 MPK and 10 MPK) showed consistent decrease amongst all molecular species of lactosylceramide.
(TIFF)

Figure S3 **Marked accumulation of lactosylceramide within cytoplasmic vesicles in mouse renal tumor cells.** Placebo mouse kidney tumor slices were stained with **A**: DAPI stain and **B**: CD-17 immunohistochemical stain for lactosylceramide.
(TIFF)

Figure S4 **Kidney histology.** **A–H**: Histology of mouse kidney sections. Red and yellow markup masks indicate areas of viable and necrotic tumor, low power images of hematoxylin & eosin stained **A**: control kidney, **B**: RENCA treated with placebo, **C**: RENCA treated with 10 MPK D-PDMP, **D**: RENCA treated with 25 MPK D-PDMP. Bar = 3 mm. High power images (200× resolution) of the above treatment groups **E–H**. **I–J**: Immunohistochemistry of sections of mouse RENCA tissue stained with antibody against PECAM-1 (CD31). **I**: Placebo demonstrates more robust CD31 staining than **J** D-PDMP (25 MPK) fed mice. **K–N**: Feeding D-PDMP decreases caspase-3 staining in mice kidney tumor. Immunohistochemical stain for Caspase-3 and corresponding digital analysis. These representative images of **K**: placebo, **L**: 10 MPK, **M**: 25 MPK, show more frequent caspase-3 positive cells in untreated mice. **N**: Quantization of caspase-3 positive cells. The percent of caspase positive cells was based on over 140,000 counted cells/treatment arm (*p<.05).
(TIFF)
