# Supplementary figures and images for: Use of a Glycolipid Inhibitor to Ameliorate Renal Cancer in a Mouse Model
Source: PLoS One. 2013 May 9;8(5):e63726. doi: 10.1371/journal.pone.0063726 (PMC3650082; doi:10.1371/journal.pone.0063726)

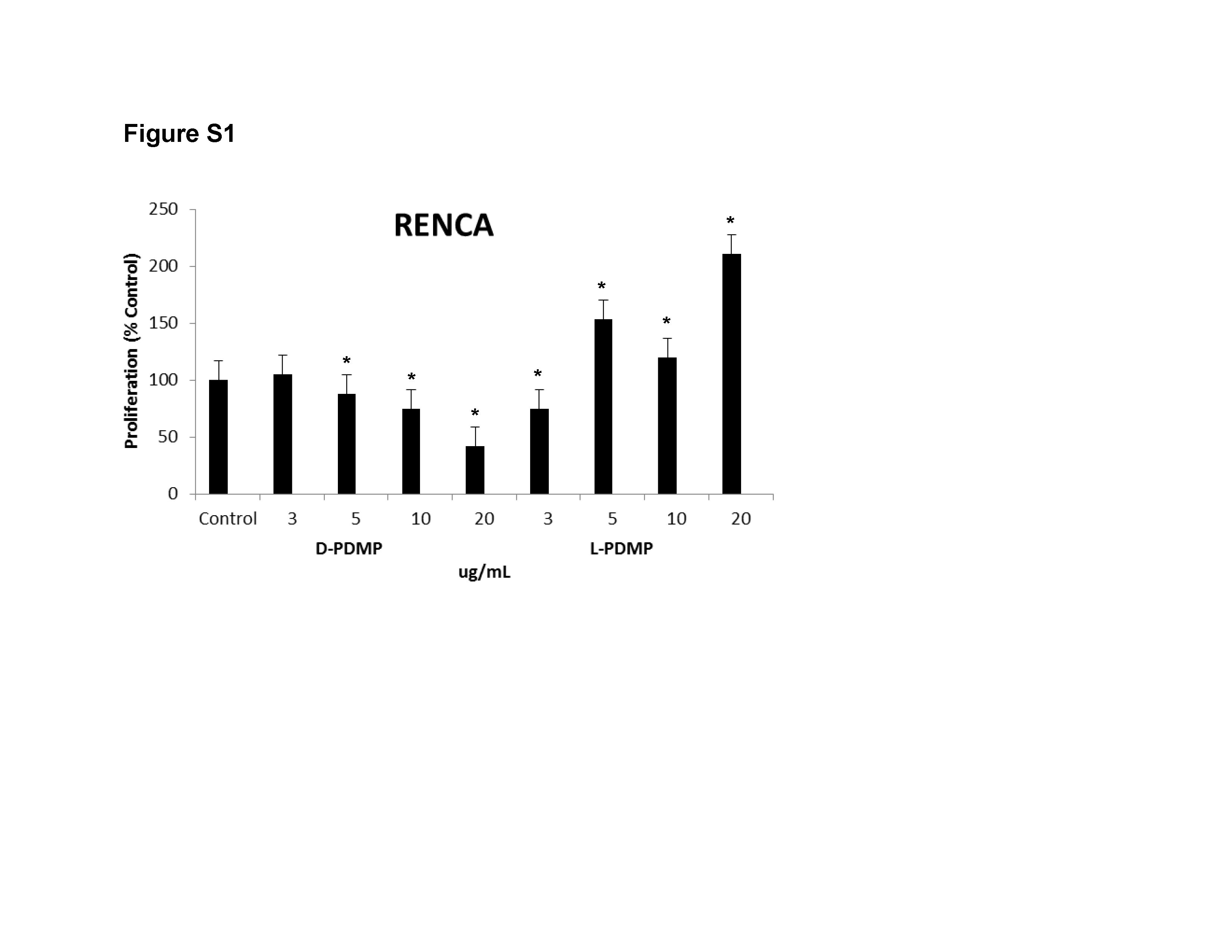

Supplement: Figure S1 — Effect of D-PDMP and L-PDMP dose on cell proliferation in mouse renal cancer cells (RENCA). Cells (×103) were seeded in 96 well sterile trays. When the cells reached ∼80% confluence, they were switched to 2% serum containing medium with various concentrations of D-PDMP and L-PDMP (3H) Thymidine (5 uci/ml). Following incubation for 24 hrs, medium was removed. Cells were washed with PBS (5×) and the incorporation of (3H) Thymidine into DNA was measured as described (N = 12, *p<.05) [12]. (TIFF) [file pone.0063726.s001.tiff]

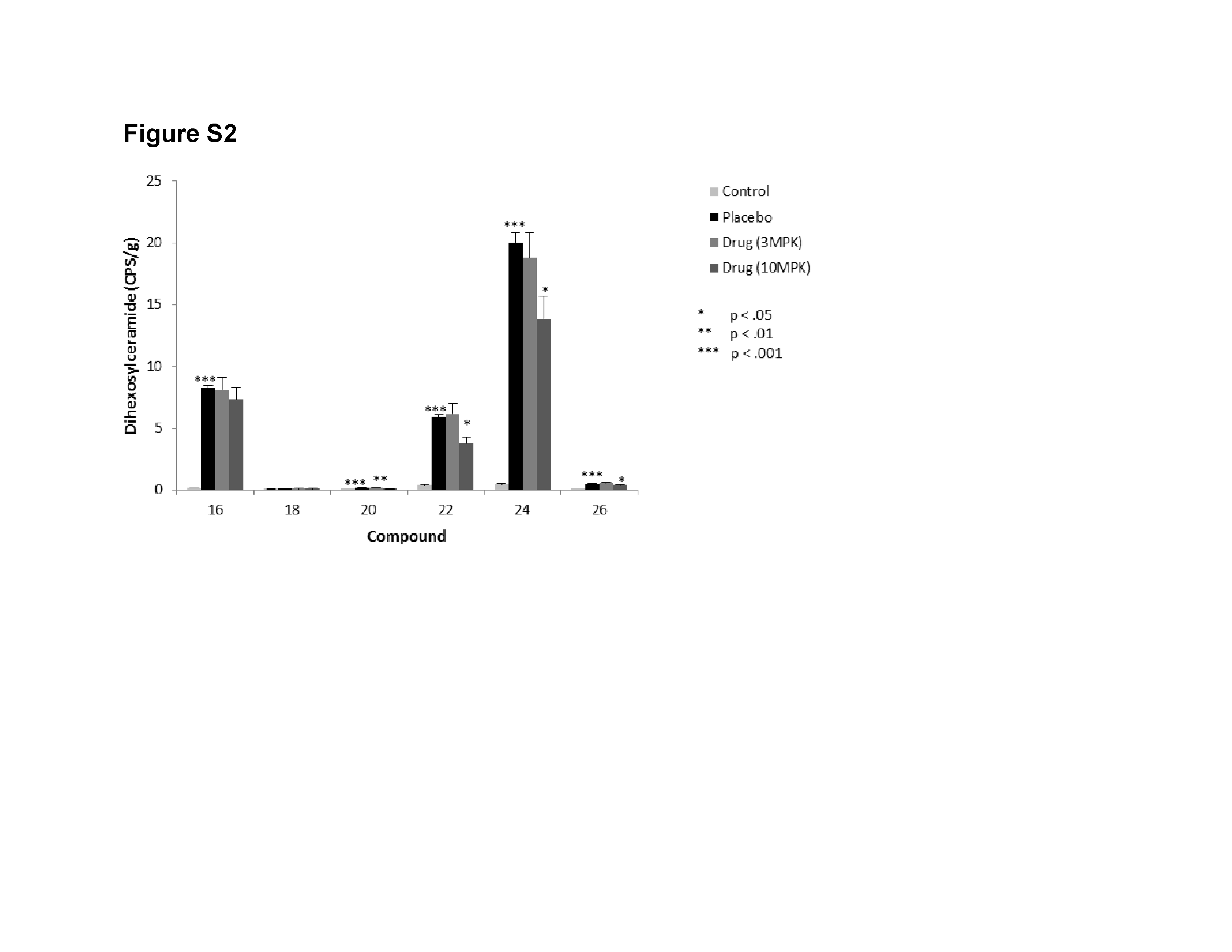

Supplement: Figure S2 — Fatty acid molecular species involved in lactosylceramide calculations. Species used for LC/MS calculation of lactosylceramide were 18∶1/16∶0, 18∶1/18∶0, 18∶1/20∶0, 18∶1/22∶0, 18∶1/24∶0, 18∶1/26∶0. Same species were used for ceramide and glucosylceramide. 18∶1/24∶0 was the most elevated species for lactosylceramide calculation. Drug treatments (3 MPK and 10 MPK) showed consistent decrease amongst all molecular species of lactosylceramide. (TIFF) [file pone.0063726.s002.tiff]

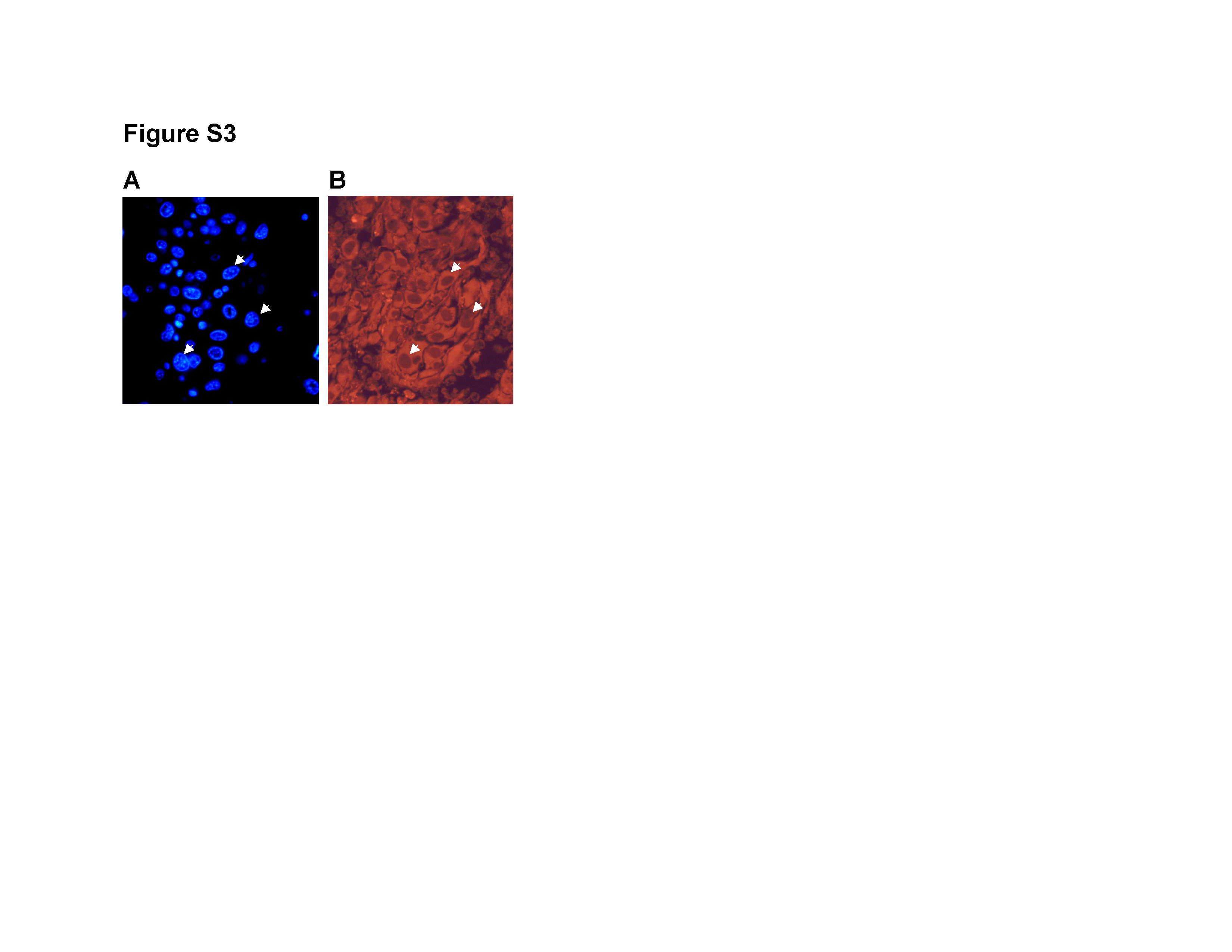

Supplement: Figure S3 — Marked accumulation of lactosylceramide within cytoplasmic vesicles in mouse renal tumor cells. Placebo mouse kidney tumor slices were stained with A: DAPI stain and B: CD-17 immunohistochemical stain for lactosylceramide. (TIFF) [file pone.0063726.s003.tiff]

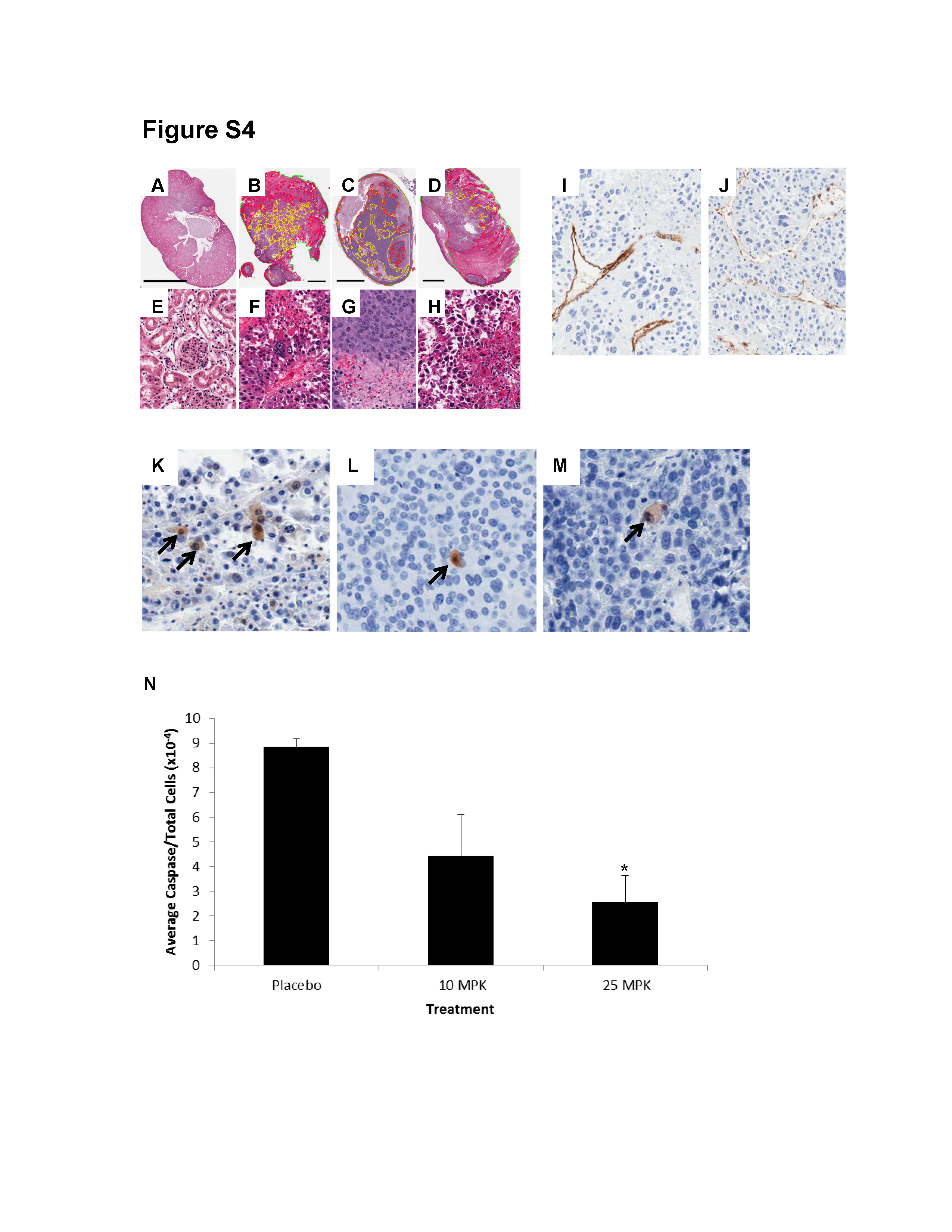

Supplement: Figure S4 — Kidney histology. A–H: Histology of mouse kidney sections. Red and yellow markup masks indicate areas of viable and necrotic tumor, low power images of hematoxylin & eosin stained A: control kidney, B: RENCA treated with placebo, C: RENCA treated with 10 MPK D-PDMP, D: RENCA treated with 25 MPK D-PDMP. Bar = 3 mm. High power images (200× resolution) of the above treatment groups E–H. I–J: Immunohistochemistry of sections of mouse RENCA tissue stained with antibody against PECAM-1 (CD31). I: Placebo demonstrates more robust CD31 staining than J D-PDMP (25 MPK) fed mice. K–N: Feeding D-PDMP decreases caspase-3 staining in mice kidney tumor. Immunohistochemical stain for Caspase-3 and corresponding digital analysis. These representative images of K: placebo, L: 10 MPK, M: 25 MPK, show more frequent caspase-3 positive cells in untreated mice. N: Quantization of caspase-3 positive cells. The percent of caspase positive cells was based on over 140,000 counted cells/treatment arm (*p<.05). (TIFF) [file pone.0063726.s004.tiff]
